# Comparative efficacy of ramosetron and ondansetron in preventing postoperative nausea and vomiting: An updated systematic review and meta-analysis with trial sequential analysis

**DOI:** 10.1371/journal.pone.0186006

**Published:** 2017-10-04

**Authors:** Ayako Yokoi, Takahiro Mihara, Koui Ka, Takahisa Goto

**Affiliations:** 1 Department of Anesthesiology, Kanagawa Children’s Medical Center, Minami-ku, Yokohama, Japan; 2 Department of Anesthesiology and Critical Care Medicine, Kanazawa-ku, Yokohama City University Graduate School of Medicine, Yokohama, Japan; International University of Health and Welfare School of Medicine, JAPAN

## Abstract

**Background:**

Postoperative nausea and vomiting is a distressing complication of surgery, and 5-HT_3_ receptor antagonists are often prescribed to prevent it. Ondansetron is the agent typically administered to prevent postoperative nausea and vomiting. Although ramosetron has a longer duration of action than ondansetron, it remains unclear whether ramosetron is the more effective medication. We performed an updated meta-analysis on the comparative efficacy of ramosetron and ondansetron in preventing postoperative nausea and vomiting.

**Methods:**

We searched six databases for all trials that randomly assigned patients to ramosetron or ondansetron groups. The primary outcome was postoperative nausea or vomiting in the early, late, and next-day periods. The secondary outcomes were side effects of the medications. We used the random-effects model to combine the results. Trial sequential analyses were performed to correct for repetitive testing in the updated meta-analysis.

**Results:**

Twenty-seven randomized controlled trials with 3,811 patients were included in the meta-analysis. The combined results of ramosetron vs. ondansetron efficacy in preventing postoperative nausea and vomiting were as follows: Risk ratio [95% confidence interval] = 0.82 [0.69–0.98] for early postoperative nausea, 0.76 [0.65–0.89] for late postoperative nausea, 0.69 [0.57–0.84] for next-day postoperative nausea, 0.78 [0.63–0.98] for early postoperative vomiting, 0.57 [0.45–0.72] for late postoperative vomiting, and 0.61 [0.43–0.86] for next-day postoperative vomiting. Dizziness was significantly lower in ramosetron groups than in ondansetron groups (risk ratio [95% confidence interval] = 0.81 [0.66–0.98]). Trial sequential analysis revealed that the results for late postoperative nausea, late postoperative vomiting, and next-day postoperative nausea were conclusive.

**Conclusions:**

Ramosetron is more effective in preventing late postoperative nausea, late postoperative vomiting, and next-day postoperative nausea than ondansetron. The incidence of dizziness may be lower in patients receiving ramosetron than in patients receiving ondansetron.

**Trial registration:**

University hospital Medical Information Network Clinical Trials Registry: UMIN000022980

## Introduction

Postoperative nausea (PON) and postoperative vomiting (POV) are common and distressing complications after surgery. The guideline [1] for the management of postoperative nausea and vomiting (PONV) recommends the use of prophylactic agents, including 5-HT_3_ receptor antagonists, for patients with a high risk of PONV.

Ramosetron is a 5-HT_3_ receptor antagonist which displays more prolonged activity than other 5-HT_3_ antagonists, such as ondansetron [2]. Previously, we reported results of the meta-analysis of the effects of ramosetron in preventing PON and POV [3]; the combined results with 637 patients (six studies) showed that ramosetron had a statistically significant effect on early POV (risk ratio [95% confidence interval] = 0.50 [0.28–0.90]) and late POV (0.53 [0.34–0.81]) but not early PON (0.79 [0.51–1.23]) and late PON (0.78 [0.60–1.46]) compared with 4 mg of ondansetron. Although ramosetron was reported to be more effective than ondasetron in preventing POV [3,4], an updated meta-analysis is required because several randomized controlled trials (RCTs) have been published since then. The Cochrane Handbook recommends that systematic reviews be updated within two years, because “systematic reviews that are not maintained may become out of date or misleading” [5]. In addition, these previous studies [3,4] looked only at RCTs which prescribed 4 mg of ondansetron. Ryu et al. reported that 8 mg of ondansetron were equally as effective as ramosetron in preventing PONV [6]. Thus, we elected to include multiple dose regimens in the current meta-analysis.

When updating a meta-analysis, the use of heterogeneity-adjusted trial sequential analysis (TSA) [7–11] is recommended because TSA-adjusted confidence intervals (CIs) can prevent inflation of the type I error rate from repetitive testing. Consequently, TSA reduces the risk of false positives in meta-analyses [7,9].

The aim of this study was to conduct an updated meta-analysis, using TSA to compare the efficacy of ramosetron and ondansetron (4-mg and 8-mg doses) in preventing PONV.

## Materials and methods

We conducted a meta-analysis following the PRISMA statement [12,13] and Cochrane Handbook [5] guidelines. Our study protocol and analysis methods were pre-specified and registered in the University hospital Medical Information Network (UMIN) Clinical Trials Registry (registration number: UMIN000022980).

### Search strategy

#### Databases searched

We searched the same databases as those included in our previous meta-analysis [3]: MEDLINE, the Cochrane Central Register of Controlled Trials, Embase, and Web of Science. The reference lists of the retrieved full articles were also searched. We then conducted a search of clinicaltrials.gov and the UMIN Clinical Trials Registry. We searched these databases on August 11, 2017.

#### Search terms and screening strategy

The search strategy was constructed by combining the following terms, as well as their synonyms: Ramosetron, postoperative, nausea, vomiting, and RCT. The details of the search strategy can be found in our previous meta-analysis [3]. To reduce screening errors, we adopted a double-check system. Two authors independently checked the titles and abstracts of RCTs identified by the initial search. Studies which were considered eligible or studies where eligibility could not be determined by checking the title or abstract were evaluated in full-text versions. The studies that met the inclusion criteria were assessed separately by two authors. Discrepancies were resolved through discussion.

### Study eligibility

We searched for all trials that randomly assigned patients to ramosetron and ondansetron groups. The exclusion criteria were the same as in our previous meta-analysis: “Trials reported by Fujii et al.; trials which did not include details of PON and/or POV; trials which did not include details of the incidence at the early, late, and/or next-day periods” [3]. We did not apply an English-language restriction.

The primary outcomes were PON and/or POV in the early, late, and next-day periods. The secondary outcomes were side effects of the medications.

The definition of the early and late period was the same as in our previous systematic review: “When the first postoperative 24 h are divided into two time periods (e.g., 0–6 hours and 6–24 h), the first time period is defined as the early period and the second time period as the late period. When the first postoperative 24 h are divided into three parts, the time period just before 6 h is defined as the early period, and the time period just after 6 h as the late period (e.g., when 24 h are divided into 0–6, 6–12, and 12–24 h, we define 0–6 h as the early period, and 6–12 h as the late period)” [3]. The next-day period was defined as 24–48 hours after surgey.

### Data abstraction

A data extraction sheet was created and included data about participants (e.g., age, American Society of Anesthesiologists [ASA] physical status), type of surgery, anesthesia use, drug treatment (dose and route of administration), primary and secondary outcomes of the study, side effects of the medications, and funding information. The data extraction strategy was the same as in our previous study: “Values originally provided as percentages were converted back into actual patient numbers for analysis. If the data were reported only in graphs which indicated percentages or numbers of patients, we measured the lengths of the graphs to obtain the percentages or numbers of patients. If ramosetron was administered by different routes or at different dosages in the same study, data with 0.3 mg of intravenous ramosetron were used because these are the most common routes and dosages” [3]. Studies in which a baseline anti-emetic was used were included, and a study evaluating baseline drug plus ramosetron treatment against baseline drug plus ondansetron treatment was counted as a ramosetron vs. ondansetron study.

If ondansetron was administered at different doses, we combined the dose groups into a single group for our primary analysis. We then conducted a subgroup analysis with an interaction analysis according to the ondansetron dose. For this purpose, the data were re-extracted when ondansetron was administered at different doses. The data in each ondansetron dose group were extracted separately, and the data from the ramosetron group were divided according to the number of ondansetron groups to avoid double-counting. Two authors extracted the data independently from the studies included and then crosschecked for discrepancies.

### Assessment of bias risk

We assessed the risk of bias as described by the Cochrane Handbook for Systematic Reviews of Interventions [5]. The following domains were assessed for bias risk: “Sequence generation”, “allocation sequence concealment”, “blinding”, “incomplete outcome data”, “selective outcome reporting”, and “other bias.” We assessed “summary risk of bias”. We assessed the summary risk of bias as “low” for RCTs with a low risk of bias in all domains; “high” for RCTs with a high risk of bias in at least one domain; “unclear” for RCTs that were neither “low” nor “high” in summary risk of bias.

### Assessment of quality of evidence

We graded the quality of evidence of the main outcomes using the Grading of Recommendations Assessment, Development, and Evaluation (GRADE) approach [14,15] with GRADEpro software (version 3.6 for Windows; available from http://ims.cochrane.org/revman/gradepro). Assessments of the quality of evidence were based on the presence or absence of the following variables: Limitations in study design, inconsistencies, indirectness, imprecision in the results, and publication bias. Evidence quality for the main outcomes was graded as very low, low, moderate, or high.

### Statistical analysis

A risk ratio (RR) with a 95% CI was used as a summary measure for dichotomous data. The random-effects model (DerSimonian and Laird method) [16] was used for combining the results of the trials. Heterogeneity was quantified with the I^2^ statistic. We considered that significant heterogeneity existed when the I^2^ statistic exceeded 50%. Small-study effects, including publication bias, were evaluated by creating a funnel plot. In addition, we applied Egger’s asymmetry test [17] to each funnel plot. Subgroup analyses were performed according to the bias risk (low vs. high or unclear) and ondansetron dose (4 mg vs. 8 mg).

TSAs [7–11] were performed to prevent false-positive or false-negative results from repetitive testing. TSA monitoring boundaries for meta-analysis and required information size (RIS) were quantified, and adjusted CIs were calculated. The RIS indicates a target sample size considering the heterogeneity of the data. To perform the TSA, we set the risk of type I error at 5% and of type II error at 10% (i.e., 90% power). The incidence of PON or POV in the control group was based on that of the included trials, and a clinically meaningful risk reduction of 25%, which was determined from a clinical perspective, was used. If the cumulative Z-curve crossed the TSA-monitoring boundary, we considered that the false-positive result rate was less than 5%. If the cumulative Z-curve crossed the futility boundary, we considered the risk ratio of ramosetron vs. ondansetron preference for the primary outcomes to be no less than 0.75, because we set a clinically meaningful anticipated relative risk reduction at 25%. If the 95% CI or the TSA-adjusted CI included a value of 1, we considered the difference to be not statistically significant. Statistical significance was set at *p* < 0.1 for the funnel plot asymmetry test.

Statistical analyses were performed using the R statistical software package, version 3.3.0 (R Foundation for Statistical Computing, Vienna, Austria). TSA was performed using TSA viewer version 0.9.5.5 β (www.ctu.dk/tsa).

## Results

### Search results

**Fig 1:**
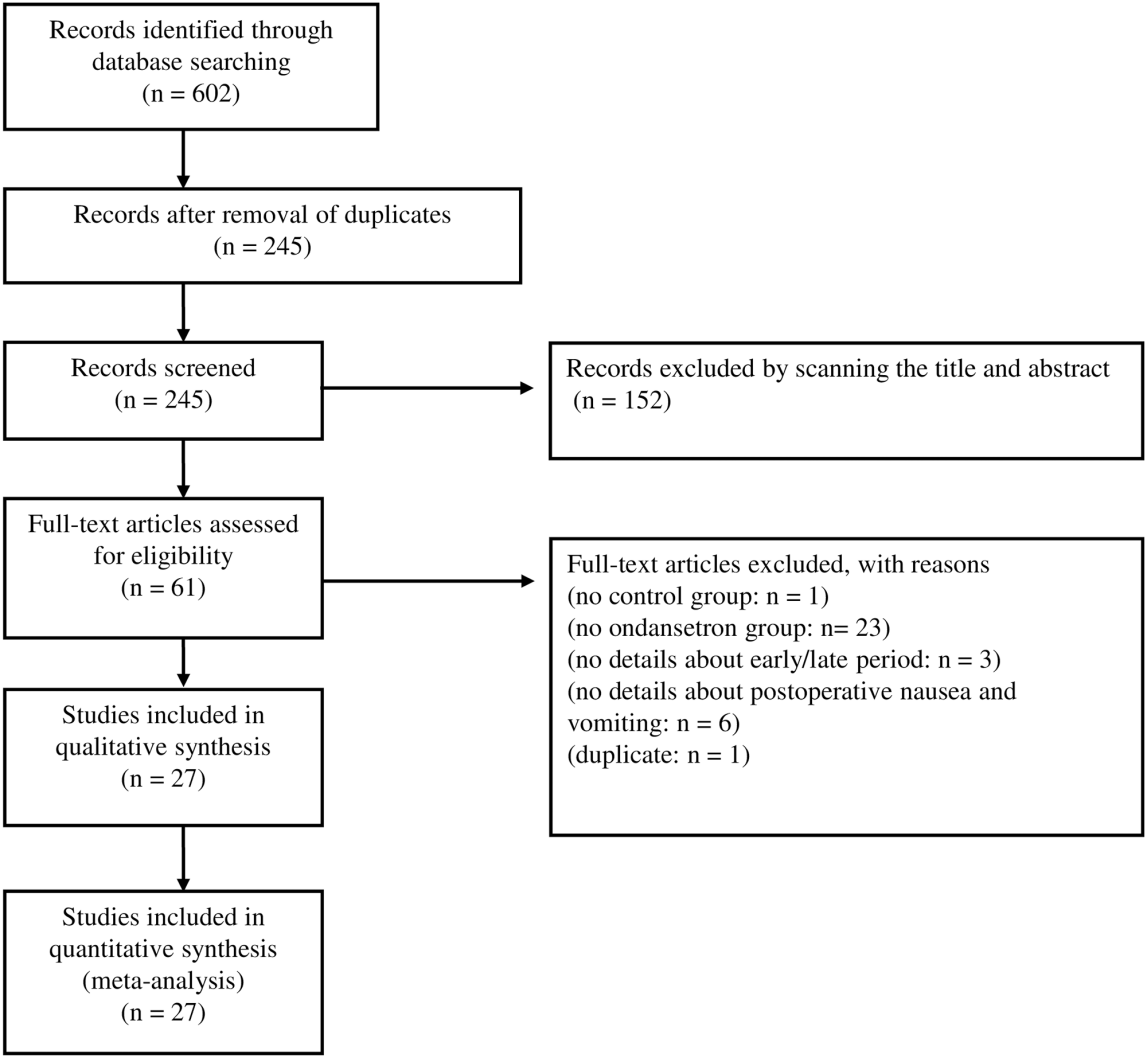
Flow diagram of the systematic review process.

Our search of MEDLINE, the Cochrane Central Register of Controlled Trials, Embase, Web of Science, clinicaltrials.gov, and the UMIN Clinical Trials Registry databases produced 602 citations. The full texts of 61 articles were examined in detail. We included 27 RCTs [6,18–43] with 3,811 patients (Fig 1). Of the 27 studies, 23 were available in English and four [32,34,36,37] in Korean. The PRISMA checklist is provided in the supporting information (S1 Table).

### Study characteristics

The details of the 27 RCTs are presented in Table 1. The participants were adults in 26 RCTs and children in one RCT [26]. Surgical procedures varied among the RCTs and included minor surgery, laparoscopic surgery, orthopedic surgery, major abdominal surgery, pulmonary lobectomy, craniotomy, and cardiac surgery. The ondansetron dose was 4 mg in 13 RCTs [18,21,24,25,27,28,30–32,37,39,41,42], 8 mg in five RCTs [19,22,33,36,40], both 4 mg and 8 mg in three RCTs [6,20,23], and other doses in six RCTs [26,29,34,35,38,43]. All RCTs used 0.3 mg of ramosetron except for four studies [26,29,34,35].

**Table 1:** Characteristics of trials included in the meta-analysis.

	ASA-PS	Age	Total number of patients	Surgery	Type of anesthesia	Route of administration	Timing of administration	Definition of early period (h)	Definition of late period (h)	Ramosetron dose	Ondansetron dose	Funding
Mujoo et al., 2017 [38]	1–2	20–60	100	elective urological procedures	N2O, isoflurane	iv	just before extubation	0–6	6–12	0.3 mg	6 mg	None
Pinsornsak et al., 2017 [40]	1–3	50–80	90	total knee arthroplasty	spinal anesthesia	iv	10 min before spinal anesthesia	0–6	6–12	0.3 mg	8 mg	None
Suman et al., 2017 [39]	1–2	18–40	100	caesarean section	spinal anesthesia	iv	after clamping of umbilical cord	0–6	6–24	0.3 mg	4 mg	Not reported
Joo et al., 2016 [41]	1–2	18–60	58	strabismus surgery	sevoflurane, remifentanil	iv	at the end of surgery	0–2	2–24	0.3 mg	4 mg	Not reported
Jamwal et al., 2016 [42]	1–2	25–55	100	Laparoscopic cholecystectomy	N2O, atracurium	iv	3 min before induction of anaesthesia	2–6	6–12	0.3 mg	4 mg	Not reported
Lee et al., 2016 [18]	1–3	Mean 53 (44–65)	1,102	gynecological or major orthopedic surgery	sevoflurane	iv	at the end of surgery	0–6	0–24	0.3 mg	4 mg	Company (Astellas Pharma Korea)
Ha et al., 2015 [19]	1–2	20–75	62	microvascular decompression with retromastoid craniotomy	sevoflurane, remifentanil	iv	at the onset of dural closure	1–6	6–24	0.3 mg	8 mg	Not reported
Shetty et al., 2015 [21]	1–2	15–60	150	middle ear surgery	general anesthesia	iv	after surgery	2–6	6–24	0.3 mg	4 mg	None
Agarkar and Chatterjee, 2015 [22]	1–2	48.2 (SD 13.6)	206	breast, parotid, thyroid, or gynecological surgery	isoflurane, N_2_O	iv	30 min before the end of surgery	0–6	6–24	0.3 mg	8 mg	Company (Cadila Healthcare Ltd.)
Shin et al., 2015 [20]	1–2	20–75	117	orthopedic surgery	spinal anesthesia	iv	5 min before spinal anesthesia	0–2	2–24	0.3 mg	4 mg and 8 mg	Company (Astellas Parma Korea)
Gupta et al., 2014 [43]	1–2	20–65	60	suregery under general anesthesia	N2O, halothane	iv	5 min prior to induction of anaesthesia	0–3	3–24	0.3 mg	0.1 mg/kg	None
Kaja et al., 2014 [24]	1–2	18–60	60	major abdominal surgeries	inhalational agent, fentanyl	iv	before extubation	0–6	6–24	0.3 mg	4 mg	None
Ryu et al., 2014 [23]	1–2	19–65	127	elective craniotomy	TIVA with propofol and remifentanil	iv	at the end of surgery	0–2	2–24	0.3 mg	4 mg and 8 mg	None
Kim et al., 2013 [25]	1–2	20–65	109	elective laparoscopic surgery	sevoflurane, remifentanil	iv	before anesthesia	0–6	6–24	0.3 mg	4 mg	Not reported
Park et al., 2013 [26]	1–2	2–15	218	elective orthopedic surgeries	sevoflurane, N_2_O	iv	at the end of surgery	0–6	6–24	6 mcg/kg	100 mcg/kg	None
Choi et al., 2012 [27]	1–2	20–65	120	lumbar spine surgery	sevoflurane	iv	at the end of surgery	0–6	6–24	0.3 mg	4 mg	Not reported
Lee et al., 2011 [28]	1–2	18–60	120	abdominal hysterectomy	sevoflurane, N_2_O	iv	at the end of surgery	0–2	2–24	0.3 mg	4 mg	Not reported
Ansari et al., 2010 [30]	1–2	30–50	130	laparoscopic cholecystectomy	sevoflurane, N_2_O	iv	at the end of surgery	NA	NA	0.3 mg	4 mg	Not reported
Choi et al., 2010 [29]	Not reported	Adult (approximately 80 years)	279	cardiac surgery	TIVA	iv	at the end of surgery	0–6	6–24	0.3 mg + 0.6 mg in PCA	4 mg + 12 mg in PCA	departmental
Hahm et al., 2010 [31]	1–3	Approximately 60 years	84	total knee replacement	CSEA + propofol (0.5–2.0 mcg/mL)	iv	at the end of surgery	0–6	6–24	0.3 mg	4 mg	None
Ryu et al., 2010 [6]	1–2	25–65	120	laparoscopic cholecystectomy	desflurane	iv	at the end of surgery	0–2	2–24	0.3 mg	4 mg and 8 mg	Not reported
Kim et al., 2009 [33]	Not reported	21–71	162	gynecological surgery	sevoflurane, N_2_O	iv	at the end of surgery	0–6	6–24	0.3 mg	8 mg	Not reported
Yoon et al., 2009 [32]	1–2	30–65	70	middle ear surgery	sevoflurane, remifentanil	iv	at the end of surgery	0–6	6–24	0.3 mg	4 mg	Not reported
Choi et al., 2008 [35]	Not reported	18–65	94	lumbar spine surgery	general anesthesia	iv	at the end of surgery	0–6	6–24	0.3 mg + saline in PCA + 0.3 mg (24 h)	4 mg + 12 mg in PCA + saline (24 h)	None
Lee et al., 2008 [34]	1–2	18–65	150	elective surgery	sevoflurane, N_2_O	oral	before induction	0–6	6–24	0.1 mg ODT	4mg + 8mg in PCA	Not reported
Suh et al., 2007 [36]	1–2	30–65	58	gynecologic surgery	sevoflurane, N_2_O	iv	at the end of surgery	0–3	3–24	0.3 mg	8 mg	Not reported
Huh et al., 2006 [37]	2–3	57.1 ± 11.6	65	lobectomy	general with epidural anesthesia	iv	at the end of surgery	0–6	6–24	0.3 mg	4 mg	Not reported

ASA, American Society of Anesthesiologists; PS, physical status; CSEA, combined spinal-epidural analgesia; NA, not available; PCA, patient-controlled analgesia; TIVA, total intravenous anesthesia; SD, standard deviation.

### Early PON

**Fig 2:**
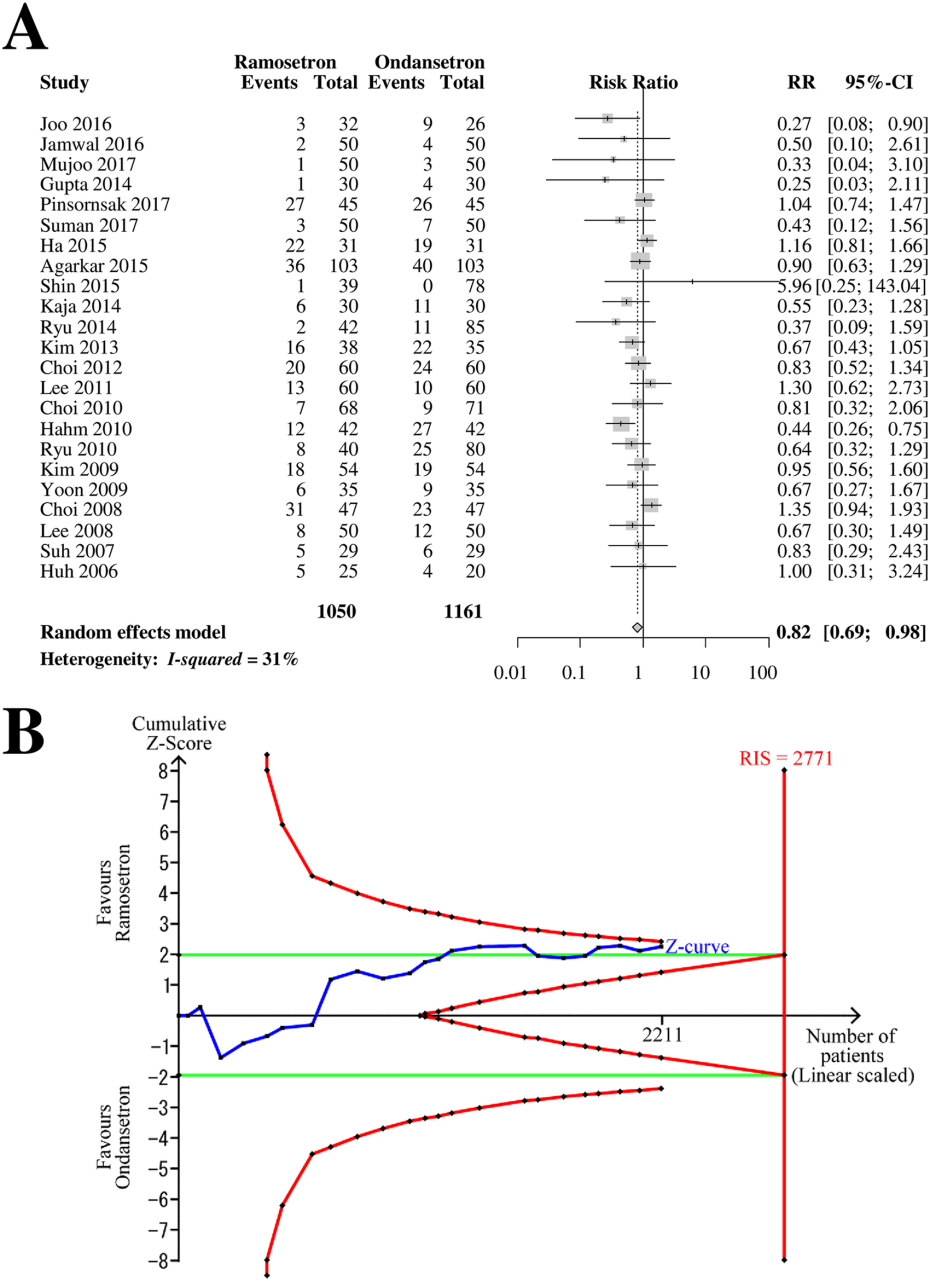
Forrest plot and trial sequential analysis of postoperative nausea (PON) in the early period. (A) Forrest plot. RR, risk ratio; CI, confidence interval. (B) Trial sequential analysis. Risk of type I error was maintained at 5% with 90% power. The variance was calculated from data obtained from the trials included in this meta-analysis. A clinically meaningful anticipated RR of the early-period PON was set at 0.75.

Twenty-three RCTs with 2,211 patients (1,050 patients in ramosetron groups and 1,161 patients in ondansetron groups) were analyzed for drug efficacy in preventing early-period PON (Fig 2). The combined results showed that ramosetron was significantly more effective in preventing early PON than ondansetron (RR [95% CI] = 0.82 [0.69–0.98], I^2^ = 31%) (Fig 2A).

When the analysis was restricted to RCTs with a low bias risk, ramosetron was more effective in preventing early PON than ondansetron (RR [95% CI] = 0.72 [0.53–0.98]). The subgroup difference according to the bias risk was not statistically significant (*p* = 0.25). The subgroup analysis revealed that there was a significant difference between 4-mg and 8-mg ondansetron treatment (*p* = 0.002, Table 2). Ramosetron was more effective in preventing early PON than 4-mg ondansetron, but similar to 8-mg ondansetron (Table 2).

**Table 2:** Ondansetron subgroup analysis.

	Summary		Ondansetron dose				
			4 mg		8 mg		*p* value for subgroup difference
	RR (95% CI)	I^2^	RR (95% CI)	I^2^	RR (95% CI)	I^2^	
Early PON	0.82 (0.69 to 0.98)	31%	0.65 (0.52 to 0.81)	0%	1.01 (0.84 to 1.21)	0%	0.002
Late PON	0.76 (0.65 to 0.89)	20%	0.68 (0.56 to 0.84)	1%	0.85 (0.65 to 1.09)	12%	0.20
Next day PON	0.69 (0.57 to 0.84)	0%	0.69 (0.55 to 0.89)	8%	0.63 (0.39 to 1.01)	0%	0.72
Early POV	0.78 (0.63 to 0.98)	0%	0.61 (0.35 to 1.06)	0%	0.87 (0.66 to 1.13)	0%	0.26
Late POV	0.57 (0.45 to 0.72)	0%	0.57 (0.35 to 0.93)	0%	0.67 (0.48 to 0.94)	0%	0.61
Next day POV	0.61 (0.43 to 0.86)	0%	0.50 (0.33 to 0.76)	0%	0.57 (0.25 to 1.31)	0%	0.76

PON, postoperative nausea; POV, postoperative vomiting; RR, risk ratio; CI, confidence interval.

The Z-curve did not cross the TSA monitoring boundary or the futility boundary (Fig 2B). The TSA-adjusted CI was 0.67–1.01, indicating the imprecision of the study. The accrued information size (n = 2,211) was 80% of the RIS (n = 2,771).

The GRADE was determined to be “low” because TSA indicated imprecise results; the funnel plot analysis, described below, indicated a suspicion of small-study effects.

### Late PON

**Fig 3:**
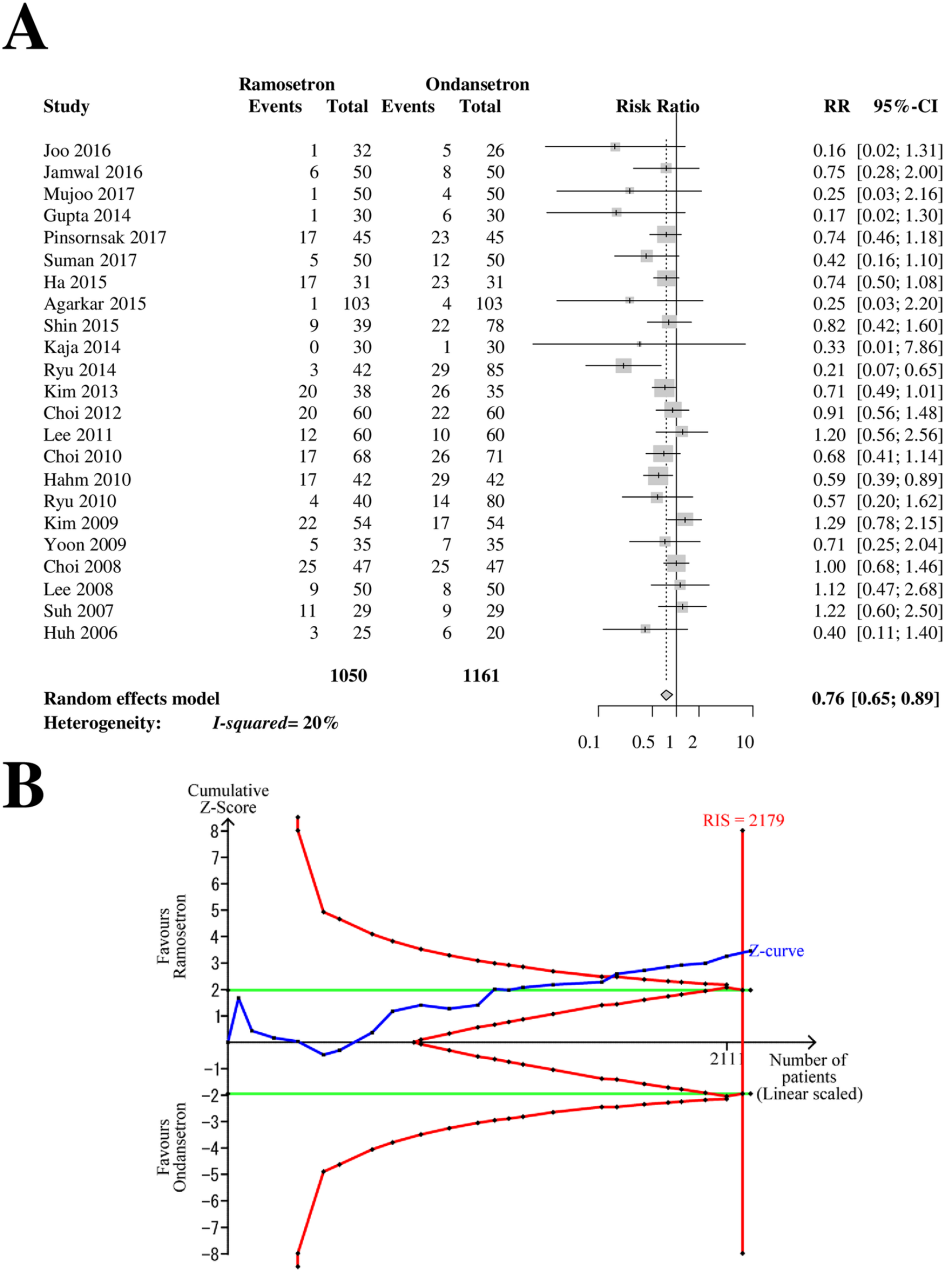
Forrest plot and trial sequential analysis of postoperative nausea (PON) in the late period. (A) Forrest plot. RR, risk ratio; CI, confidence interval. (B) Trial sequential analysis. Risk of type I error was maintained at 5% with 90% power. The variance was calculated from data obtained from the trials included in this meta-analysis. A clinically meaningful anticipated RR of the late-period PON was set at 0.75.

Twenty-three RCTs with 2,211 patients (1,050 patients in ramosetron groups and 1,161 patients in ondansetron groups) were analyzed for drug efficacy in preventing late-period PON (Fig 3). The combined results showed that ramosetron was significantly more effective at preventing late PON than ondansetron (RR [95% CI] = 0.76 [0.65–0.89], I^2^ = 20%) (Fig 3A).

When the analysis was restricted to RCTs with a low risk of bias, it yielded a similar result (RR [95% CI] = 0.69 [0.50–0.97]). The subgroup difference according to the bias risk was not statistically significant (*p* = 0.43). The subgroup analysis revealed that the difference in efficacy between 4-mg and 8-mg ondansetron was not statistically significant (*p* = 0.20, Table 2).

The Z-curve crossed the TSA monitoring boundary (Fig 3B). The TSA-adjusted CI was 0.64–0.90. The accrued information size (n = 2,211) reached the RIS (n = 2,179).

The GRADE was determined to be “moderate” because the funnel plot analysis, described below, indicated a suspicion of small-study effects.

### Next-day PON

**Fig 4:**
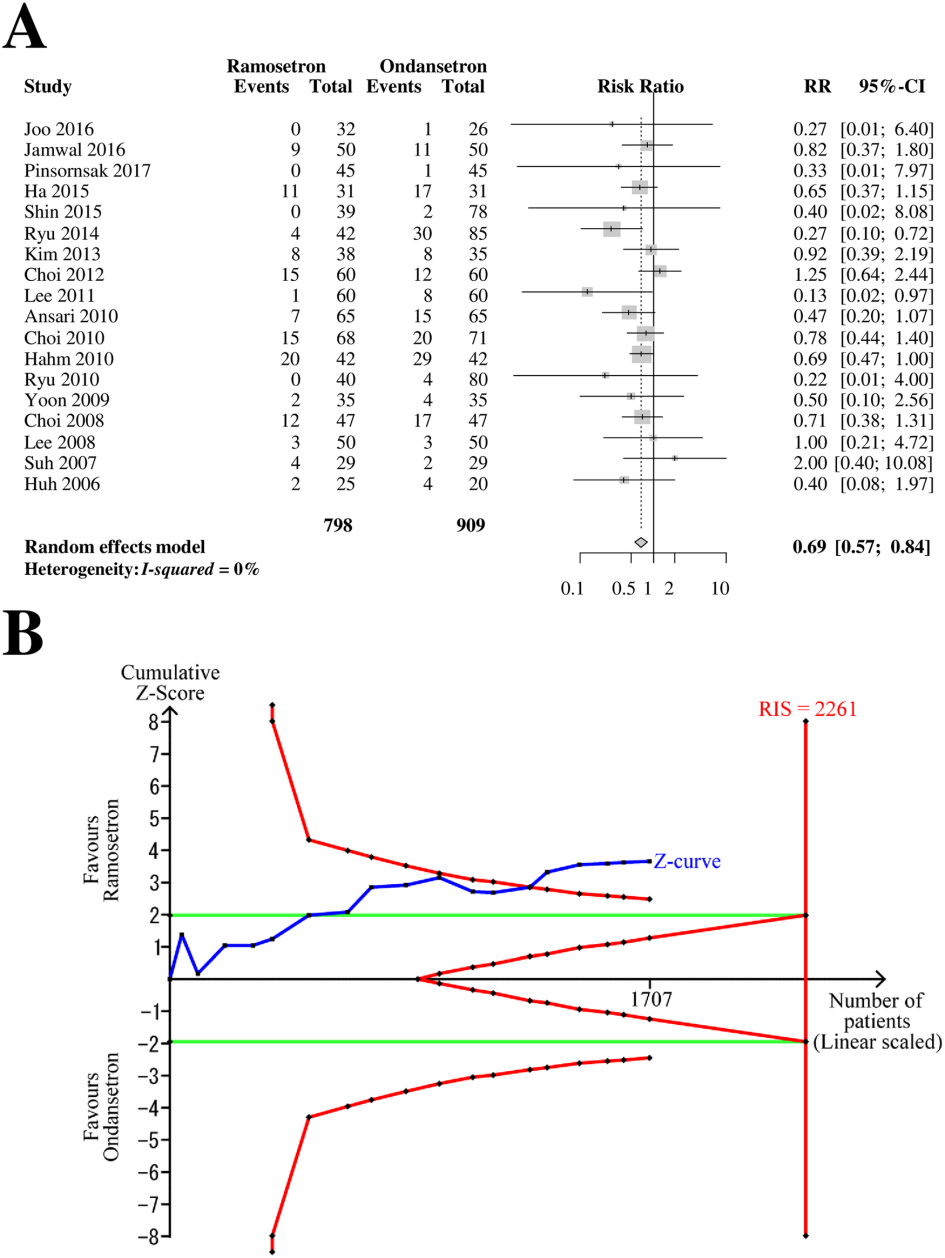
Forrest plot and trial sequential analysis of postoperative nausea (PON) in the next-day period. (A) Forrest plot. RR, risk ratio; CI, confidence interval. (B) Trial sequential analysis. Risk of type I error was maintained at 5% with 90% power. The variance was calculated from data obtained from the trials included in this meta-analysis. A clinically meaningful anticipated RR of the next-day period PON was set at 0.75.

Eighteen RCTs with 1,707 patients (798 patients in ramosetron groups and 909 patients in ondansetron groups) were analyzed for ramosetron and ondansetron efficacy in preventing next-day PON (Fig 4). The combined results showed that ramosetron had significantly greater efficacy in preventing next-day PON than ondansetron (RR [95% CI] = 0.69 [0.57–0.84], I^2^ = 0%) (Fig 4A).

When the analysis was restricted to RCTs with a low risk of bias, it yielded a similar result (RR [95% CI] = 0.59 [0.36–0.95]). The subgroup difference according to the bias risk was not statistically significant (*p* = 0.43). The subgroup analysis revealed that the difference between 4-mg and 8-mg ondansetron was not statistically significant (*p* = 0.72, Table 2).

The Z-curve crossed the TSA monitoring boundary (Fig 4B). The TSA-adjusted CI was 0.54–0.89. The accrued information size (n = 1,707) was 75% of the RIS (n = 2,261).

The GRADE was determined to be “high” because there was no inconsistency, imprecision, indirectness, suspicion of biased result from high or unclear risk of bias, or suspicion of publication bias.

### Early POV

**Fig 5:**
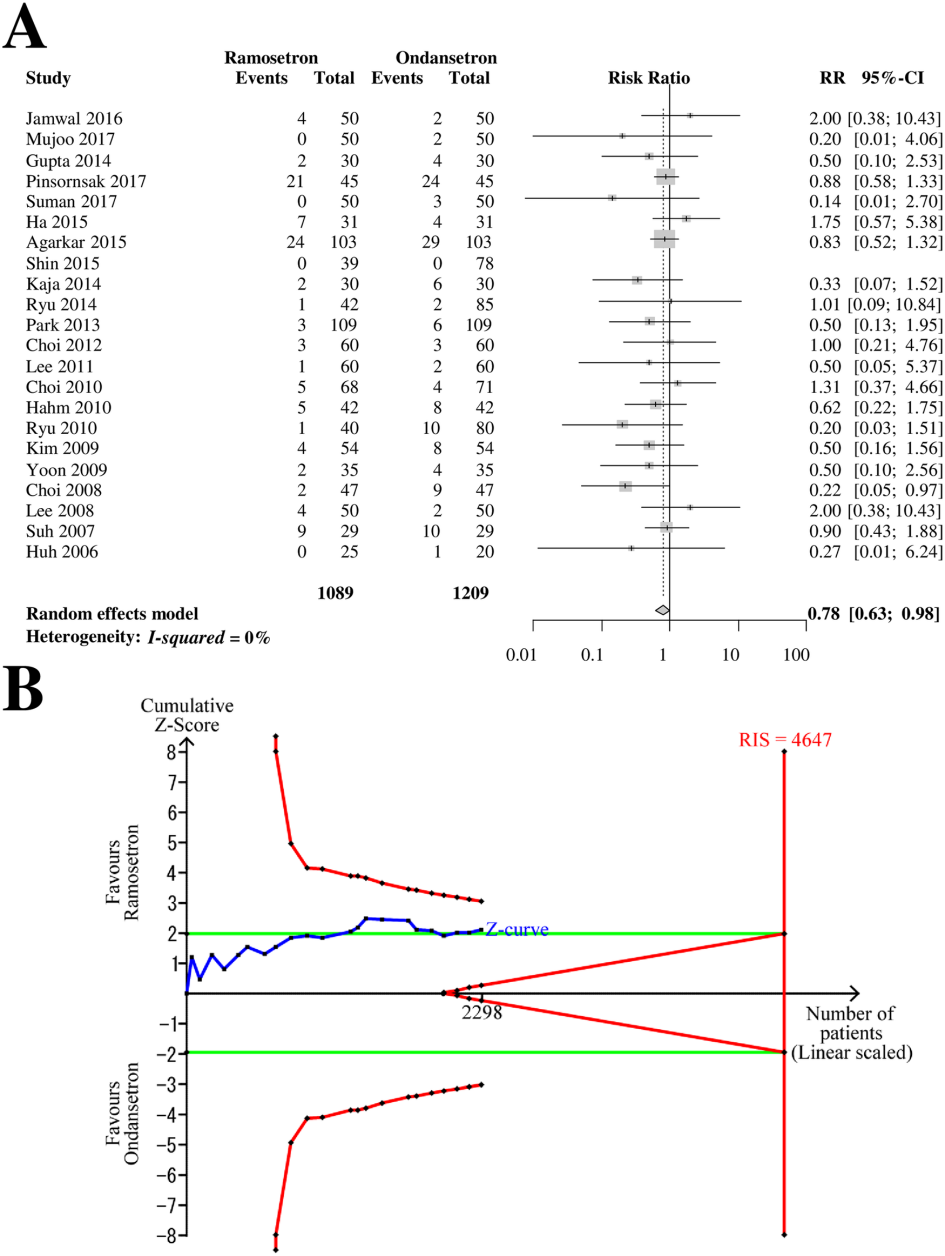
Forrest plot and trial sequential analysis of postoperative vomiting (POV) in the early period. (A) Forrest plot. RR, risk ratio; CI, confidence interval. (B) Trial sequential analysis. Risk of type I error was maintained at 5% with 90% power. The variance was calculated from data obtained from the trials included in this meta-analysis. A clinically meaningful anticipated RR of the early-period POV was set at 0.75.

Twenty-two RCTs with 2,298 patients (1,089 patients in ramosetron groups and 1,209 patients in ondansetron groups) were analyzed for ramosetron and ondansetron efficacy in preventing early-period POV (Fig 5). The combined results showed that ramosetron was significantly more effective in preventing early POV than ondansetron (RR [95% CI] = 0.78 [0.63–0.98], I^2^ = 0%) (Fig 5A).

When the analysis was restricted to RCTs with a low bias risk, it yielded a similar result, but the 95% CI widened (RR [95% CI] = 0.76 [0.54–1.09]). The subgroup difference according to the bias risk was not statistically significant (*p* = 0.86). The subgroup analysis revealed that the difference in efficacy between 4-mg and 8-mg ondansetron was not statistically significant (*p* = 0.26, Table 2).

The Z-curve did not cross the TSA monitoring boundary or the futility boundary (Fig 5B). The TSA-adjusted CI was 0.55–1.11. The accrued information size (n = 2,298) was 49% of the RIS (n = 4,647).

The GRADE was determined to be “low” because the TSA indicated that the result was imprecise.

### Late POV

**Fig 6:**
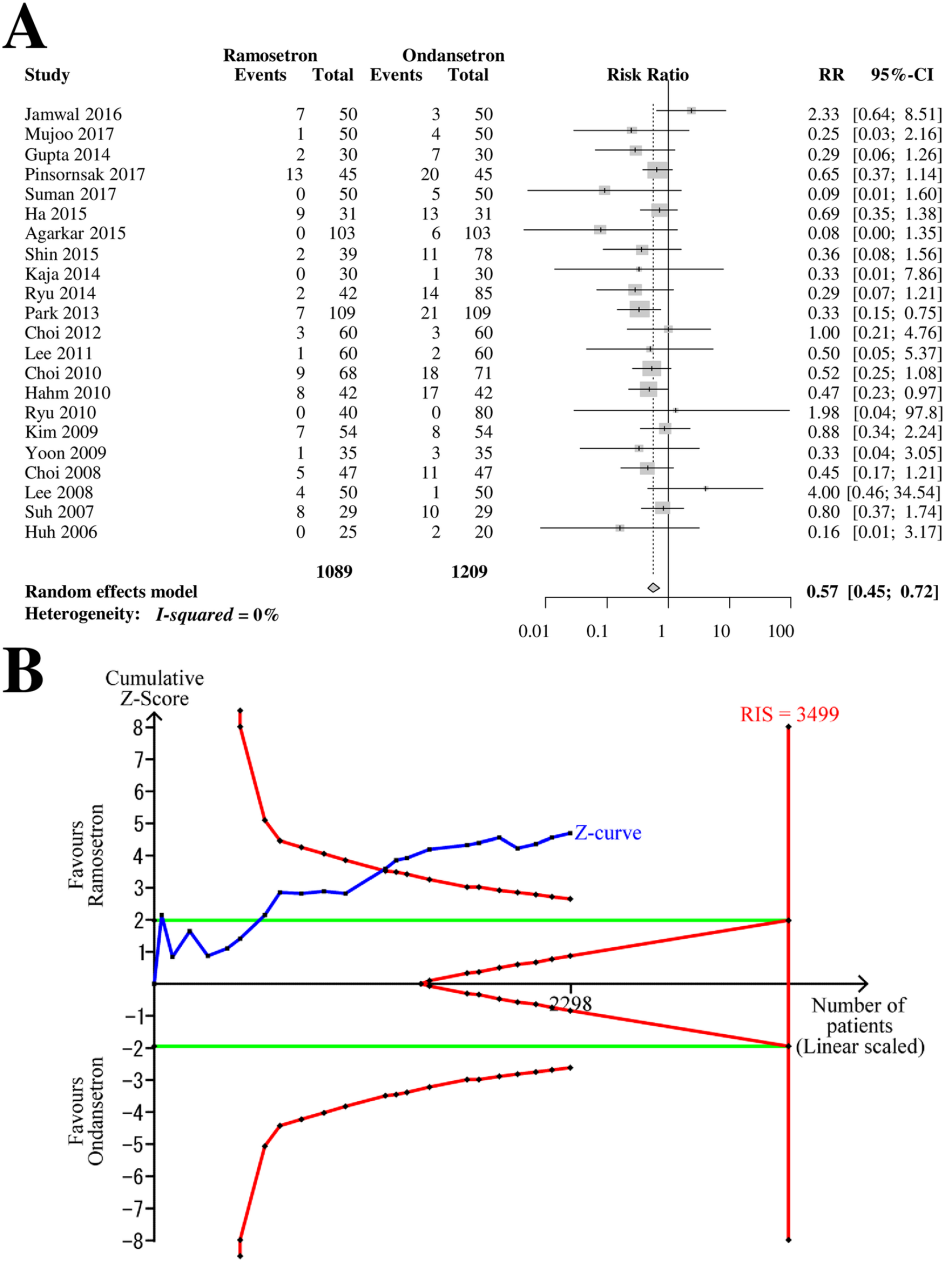
Forrest plot and trial sequential analysis of postoperative vomiting (POV) in the late period. (A) Forrest plot. RR, risk ratio; CI, confidence interval. (B) Trial sequential analysis. Risk of type I error was maintained at 5% with 90% power. The variance was calculated from data obtained from the trials included in this meta-analysis. A clinically meaningful anticipated RR of the late-period POV was set at 0.75.

Twenty-two RCTs with 2,298 patients (1,089 patients in ramosetron groups and 1,209 patients in ondansetron groups) were analyzed for ramosetron and ondansetron efficacy in preventing late-period POV (Fig 6). The combined results showed that ramosetron was significantly more effective in preventing late-period POV than ondansetron (RR [95% CI] = 0.57 [0.45–0.72], I^2^ = 0%) (Fig 6A).

When the analysis was restricted to RCTs with low bias risk, it yielded a similar result (RR [95% CI] = 0.59 [0.40–0.87]). The subgroup difference according to the bias risk was not statistically significant (*p* = 0.80). The subgroup analysis revealed that the difference in efficacy between 4-mg and 8-mg ondansetron was not statistically significant (*p* = 0.61, Table 2).

The Z-curve crossed the TSA monitoring boundary (Fig 6B). The TSA-adjusted CI was 0.42–0.78. The accrued information size (n = 2,298) was 66% of the RIS (n = 3,499).

The GRADE was determined to be “high” because there was no inconsistency, imprecision, indirectness, suspicion of biased result from high or unclear risk of bias, or suspicion of publication bias.

### Next-day POV

**Fig 7:**
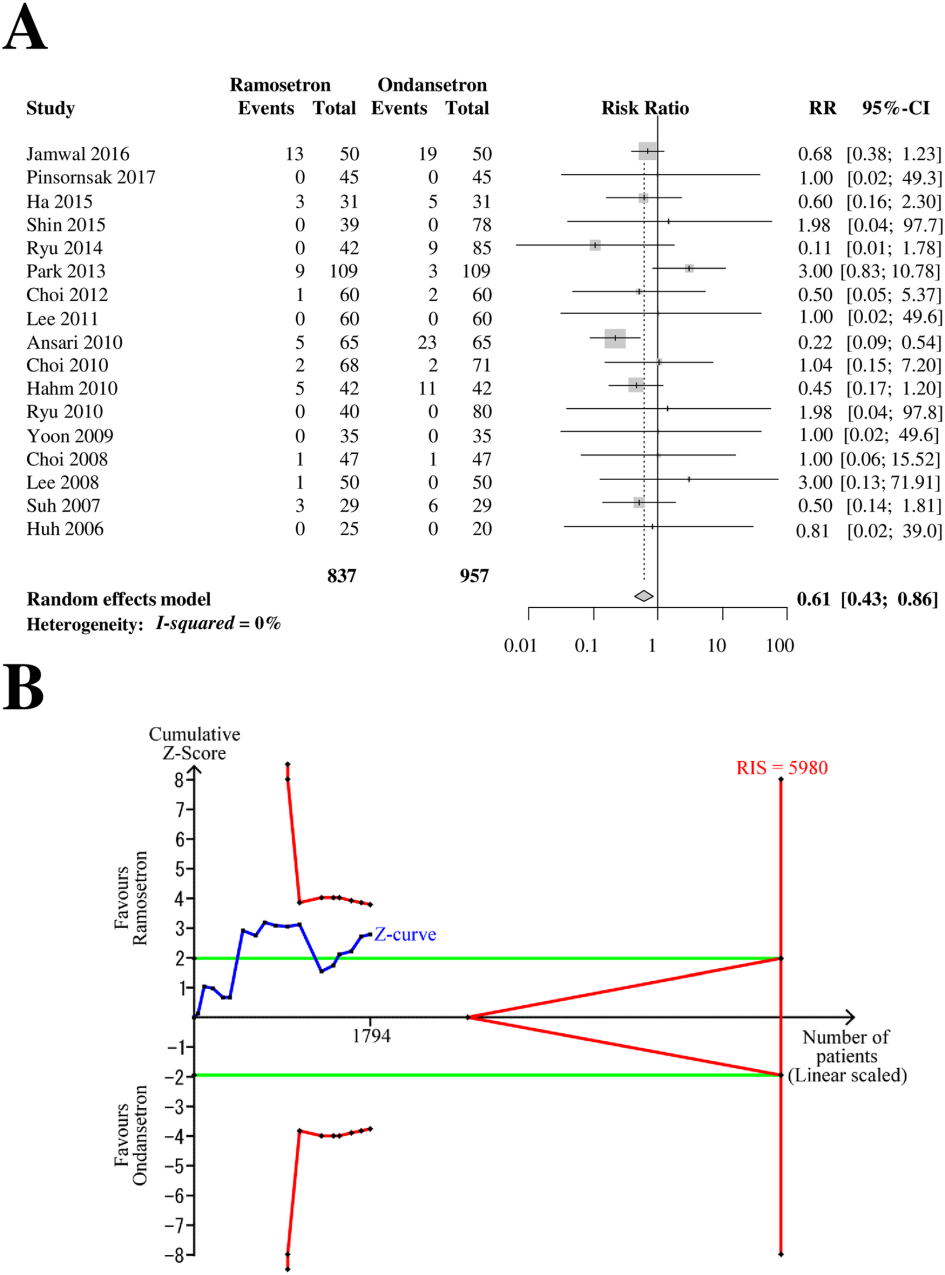
Forrest plot and trial sequential analysis of postoperative vomiting (POV) in the next-day period. (A) Forrest plot. RR, risk ratio; CI, confidence interval. (B) Trial sequential analysis. Risk of type I error was maintained at 5% with 90% power. The variance was calculated from data obtained from the trials included in this meta-analysis. A clinically meaningful anticipated RR of the next-day period POV was set at 0.75.

Seventeen RCTs with 1,794 patients (837 patients in ramosetron groups and 957 patients in ondansetron groups) were analyzed for ramosetron and ondansetron efficacy in preventing next-day POV (Fig 7). The combined results showed that ramosetron was significantly more effective in preventing next-day POV than ondansetron (RR [95% CI] = 0.61 [0.43–0.86], I^2^ = 0%) (Fig 7A).

When the sensitivity analysis was restricted to RCTs with low bias risk, it yielded a similar result, but the 95% CI widened (RR [95% CI] = 0.44 [0.19–1.05]). The subgroup difference according to the bias risk was not statistically significant (*p* = 0.43). The subgroup analysis revealed that the difference in efficacy between 4-mg and 8-mg ondansetron was not statistically significant (*p* = 0.76, Table 2).

The Z-curve did not cross the TSA monitoring boundary or the futility boundary (Fig 7B). The TSA-adjusted CI was 0.31–1.19. The accrued information size (n = 1,794) was 30% of the RIS (n = 5,980).

The GRADE was determined to be “low” because the TSA indicated that the result was imprecise.

### Side effects

**Fig 8:**
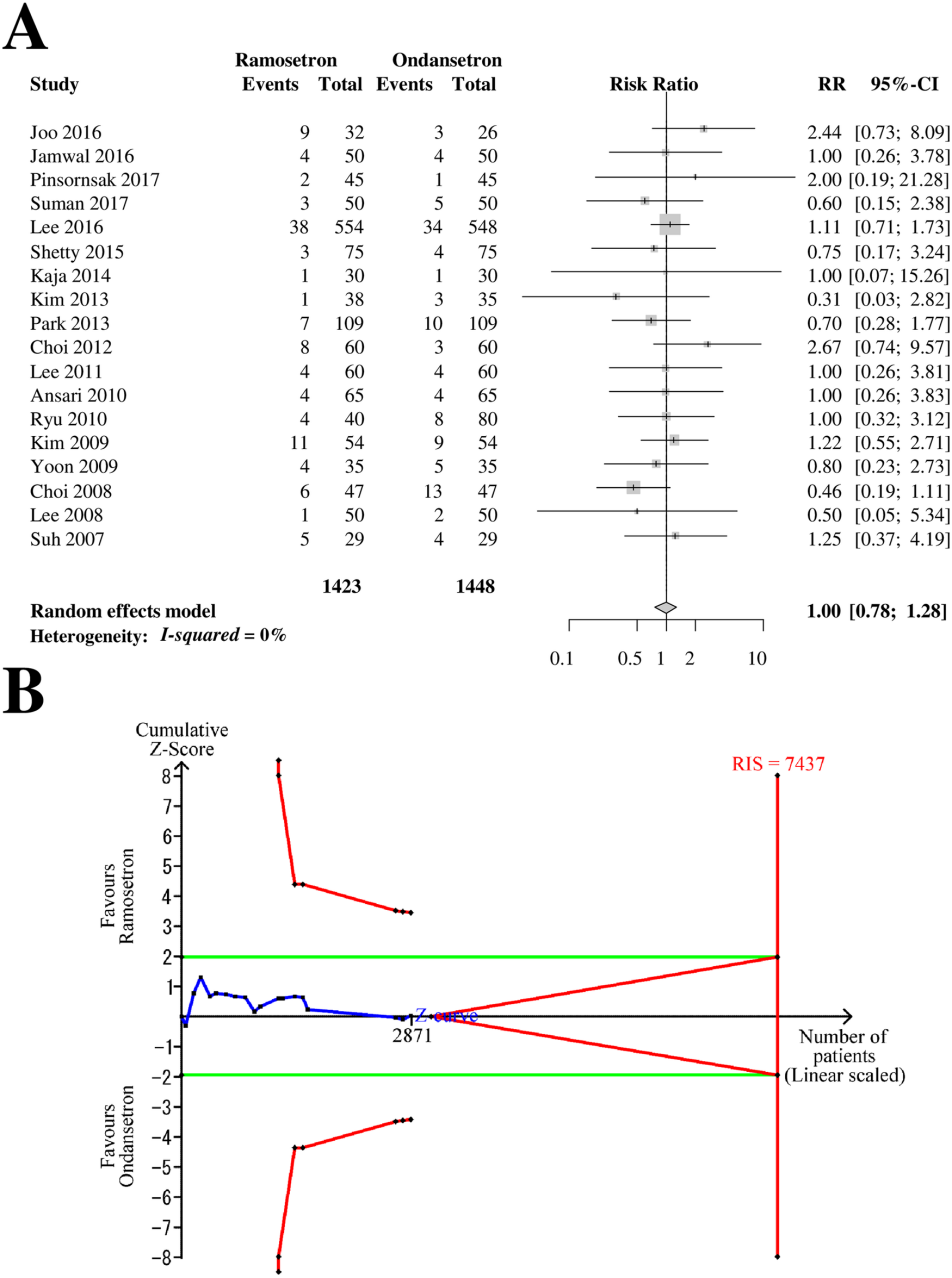
Forrest plot and trial sequential analysis for headache. (A) Forrest plot. RR, risk ratio, CI, confidence interval. (B) Trial sequential analysis. Risk of type I error was maintained at 5% with 90% power. The variance was calculated from data obtained from the trials included in this meta-analysis. A clinically meaningful anticipated RR of the early-period postoperative nausea was set at 0.75.

Eighteen RCTs with 2,871 patients (1,423 patients in ramosetron groups and 1,448 patients in ondansetron groups) were analyzed for headache as a side effect (Fig 8). The combined results showed that the incidence of headache did not differ significantly between ramosetron and ondansetron treatment groups (RR [95% CI] = 1.00 [0.78–1.28], I^2^ = 0%) (Fig 8A). The subgroup analysis revealed that the differences according to bias risk and ondansetron dose (4 mg vs. 8 mg) were not statistically significant (*p* = 0.79 and 0.73, respectively). The Z-curve did not cross the TSA monitoring boundary or the futility boundary (TSA-adjusted CI = 0.67–1.49, Fig 8B). The GRADE was determined to be “low” because the TSA indicated that the result was imprecise.

**Fig 9:**
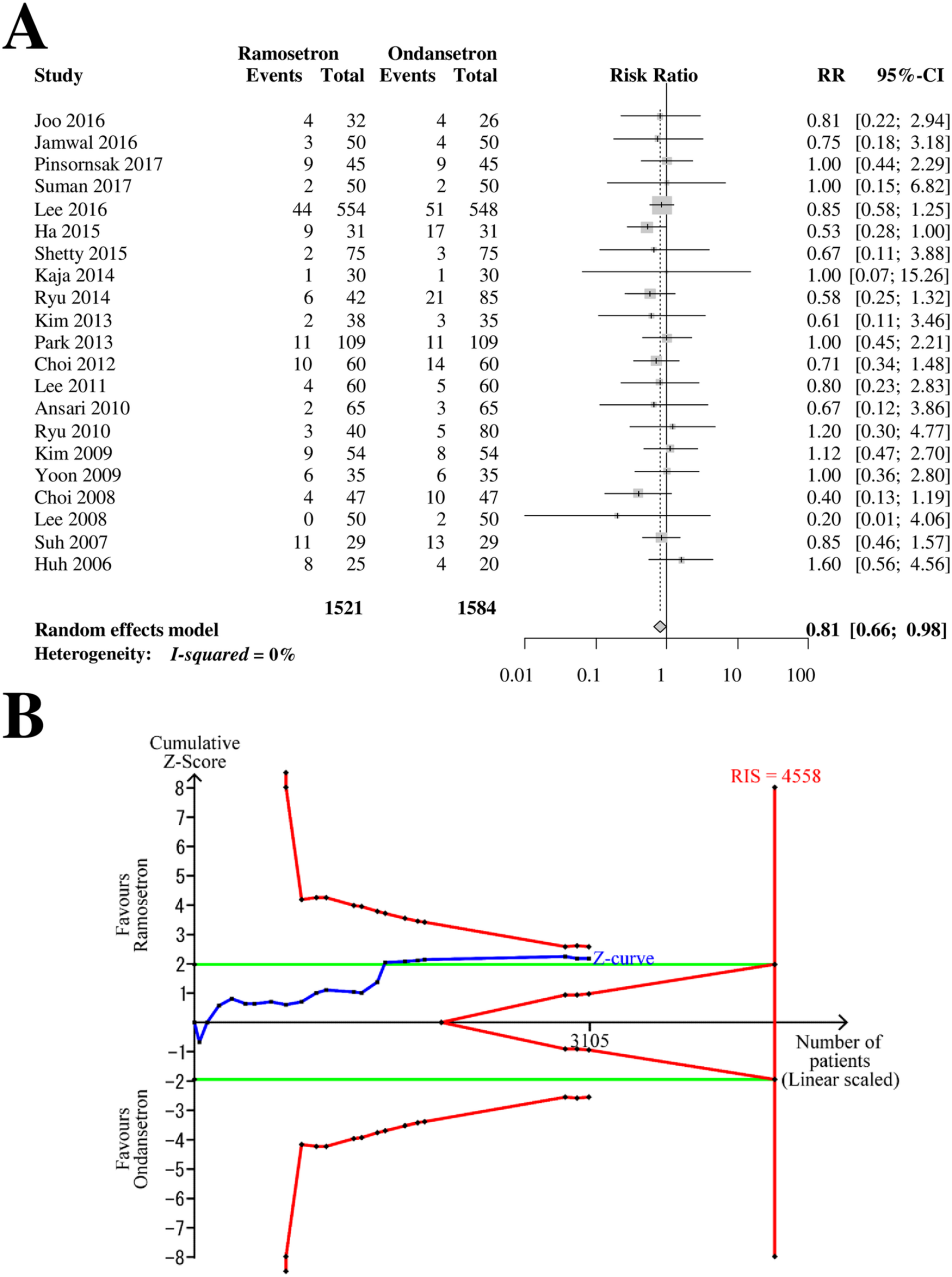
Forrest plot and trial sequential analysis for dizziness. (A) Forrest plot. RR, risk ratio; CI, confidence interval. (B) Trial sequential analysis. Risk of type I error was maintained at 5% with 90% power. The variance was calculated from data obtained from the trials included in this meta-analysis. A clinically meaningful anticipated RR of the early-period postoperative nausea was set at 0.75.

Twenty-one RCTs with 3,105 patients (1,521 patients in ramosetron groups and 1,584 patients in ondansetron groups) were analyzed for dizziness as a side effect (Fig 9). The combined results showed that the incidence of dizziness was lower in patients receiving ramosetron than in patients receiving ondansetron (RR [95% CI] = 0.81 [0.66–0.98], I^2^ = 0%) (Fig 9A). The subgroup analysis revealed that the differences according to bias risk and ondansetron dose (4 mg vs. 8 mg) were not statistically significant (*p* = 0.71 and 0.60, respectively). The Z-curve did not cross the TSA monitoring boundary or the futility boundary (TSA-adjusted CI = 0.62–1.04, Fig 9B). The GRADE was determined to be “low” because the TSA indicated that the result was imprecise.

**Fig 10:**
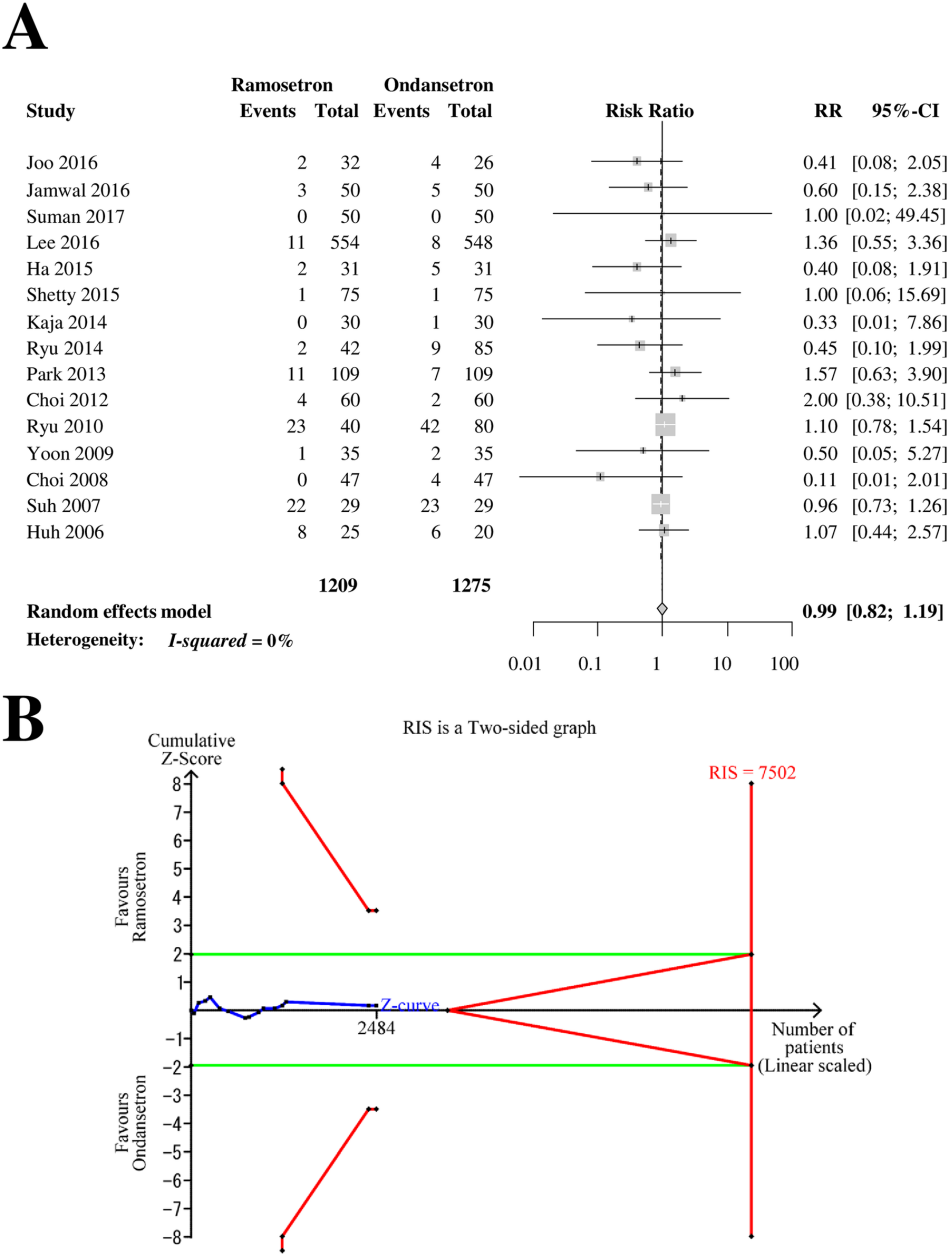
Forrest plot and trial sequential analysis for drowsiness. (A) Forrest plot. RR, risk ratio; CI, confidence interval. (B) Trial sequential analysis. Risk of type I error was maintained at 5% with 90% power. The variance was calculated from data obtained from the trials included in this meta-analysis. A clinically meaningful anticipated RR of the early-period postoperative nausea was set at 0.75.

Fifteen RCTs with 2,484 patients (1,209 patients in ramosetron groups and 1,275 patients in ondansetron groups) were analyzed for drowsiness as a side effect (Fig 10). The combined results showed that the incidence of drowsiness did not differ significantly between ramosetron and ondansetron treatment groups (RR [95% CI] = 0.99 [0.82–1.19], I^2^ = 0%) (Fig 10A). The subgroup analysis revealed that the differences according to bias risk and ondansetron dose (4 mg vs. 8 mg) were not statistically significant (*p* = 0.93 and 0.68, respectively). The Z-curve did not cross the TSA monitoring boundary or the futility boundary (TSA-adjusted CI = 0.72–1.35, Fig 10B). The GRADE was determined to be “low” because the TSA indicated that the result was imprecise.

### Small-study effects

**Fig 11:**
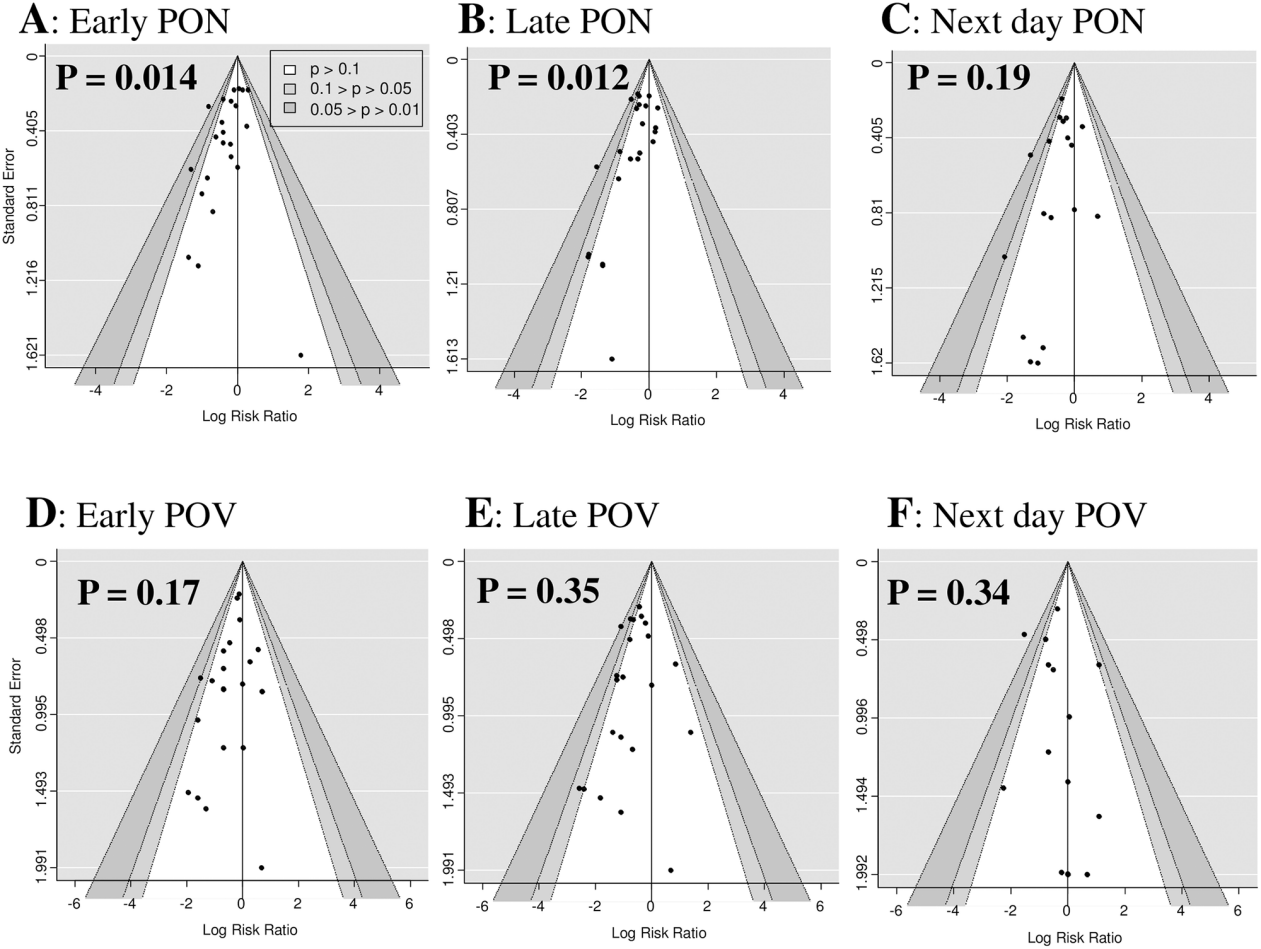
Funnel plots for postoperative nausea (PON) or vomiting (POV) in early, late, and next-day periods.

The funnel plots of the primary outcomes are shown in Fig 11. The asymmetry tests for these funnel plots were significant for early PON (*p* = 0.014) and late PON (*p* = 0.012), but not significant for next-day PON (*p* = 0.19), early POV (*p* = 0.17), late POV (*p* = 0.35), and next-day POV (*p* = 0.34). The asymmetry tests for headache (*p* = 0.71), dizziness (*p* = 0.81) and drowsiness (*p* = 0.18) were also non-significant.

### Risk of bias

The assessments of the bias risk in individual RCTs are shown in Table 3. There were six RCTs [6,23,25,31,33,40] with low bias risks in all domains. While 17 double-blinded RCTs should have given a low bias risk, the allocation concealment was unclear in 10 of them (Table 3).

**Table 3:** Risk of bias in individual trials.

	Sequence generation	Allocation consealment	Patients blinded	Health care providers blinded	Data collectors blinded	outcome assessors blinded	imcomplete outcome data	Selective reporting	other bias	summary
Mujoo et al., 2017 [38]	Low	Unclear	Low	Low	Low	Low	Low	Unclear	Low	Unclear
Pinsornsak et al., 2017 [40]	Low	Low	Low	Low	Low	Low	Low	Low	Low	Low
Suman et al., 2017 [39]	Low	Unclear	Unclear	Unclear	Unclear	Unclear	Low	Low	Unclear	Unclear
Joo et al., 2016 [41]	Low	Low	Low	Low	Low	Low	Unclear	Unclear	Unclear	Unclear
Jamwal et al., 2016 [42]	Unclear	Unclear	Low	High	High	High	Low	Low	Unclear	High
Lee et al., 2016 [18]	Low	Unclear	Low	Unclear	Low	Low	Unclear	Low	High	High
Ha et al., 2015 [19]	Low	Unclear	Low	Low	Low	Low	Low	Low	Low	Unclear
Shetty et al., 2015 [21]	Low	Low	Low	Unclear	Unclear	Unclear	Low	High	Unclear	High
Agarkar and Chatterjee, 2015 [22]	Low	Unclear	Low	Low	Unclear	Unclear	Low	Low	Low	Unclear
Shin et al., 2015 [20]	Low	Unclear	Low	Low	Low	Low	Low	Low	Low	Unclear
Gupta et al., 2014 [43]	Unclear	Low	Low	Low	Low	Low	Low	Unclear	Low	Unclear
Kaja et al., 2014 [24]	Low	Unclear	Low	Unclear	Unclear	Unclear	Low	Low	Low	Unclear
Ryu et al., 2014 [23]	Low	Low	Low	Low	Low	Low	Low	Low	Low	Low
Kim et al., 2013 [25]	Low	Low	Low	Low	Low	Low	Low	Low	Low	Low
Park et al., 2013 [26]	Low	Unclear	Low	Low	Low	Low	Low	Low	Low	Unclear
Choi et al., 2012 [27]	Low	Unclear	Low	Low	Unclear	Unclear	Low	Low	Low	Unclear
Lee et al., 2011 [28]	Low	Unclear	Low	Low	Low	Low	Low	Low	Low	Unclear
Ansari et al., 2010 [30]	Low	Unclear	Low	Low	Low	Low	Low	Low	Low	Unclear
Choi et al., 2010 [29]	Low	Unclear	Low	Low	Low	Low	Low	Low	Unclear	Unclear
Hahm et al., 2010 [31]	Low	Low	Low	Low	Low	Low	Low	Low	Low	Low
Ryu et al., 2010 [6]	Low	Low	Low	Low	Low	Low	Low	Low	Low	Low
Kim et al., 2009 [33]	Low	Low	Low	Low	Low	Low	Low	Low	Low	Low
Yoon et al., 2009 [32]	Unclear	Unclear	Low	Unclear	Low	Low	Low	Low	Low	Unclear
Choi et al., 2008 [35]	Low	Unclear	Low	Low	Low	Low	Low	Low	Low	Unclear
Lee et al., 2008 [34]	Unclear	Unclear	High	High	Unclear	Unclear	Low	Low	Low	High
Suh et al., 2007 [36]	Unclear	Unclear	Low	High	Low	Low	Low	Low	Low	High
Huh et al., 2006 [37]	Unclear	Unclear	Low	Unclear	Low	Low	Low	Low	Low	Unclear

## Discussion

The findings of this updated meta-analysis were as follows: 1) Ramosetron was more effective in preventing late-period PON, late-period POV, and next-day PON than ondansetron; 2) the effect of ramosetron in preventing early-period PON may be greater than that of 4-mg ondansetron, but not of 8-mg ondansetron; 3) the incidence of dizziness may be significantly lower in patients receiving ramosetron than in patients receiving ondansetron, but the incidences of headache and drowsiness were similar in patients in both drug groups.

Our results indicate that ramosetron is more effective in preventing PON and POV in late (6–24 h) and next-day (24–48 h) periods than ondansetron. The GRADE was “moderate” for late PON and “high” for late POV and next-day PON. These findings could be explained by the longer duration of action of ramosetron. The elimination half-life of ramosetron is 9 h [2] whereas that of ondansetron is 3.5 h [44]. Therefore, ramosetron offered prolonged benefits in late or next-day periods. A previous observational study [45] reported that the incidence of POV or moderate-severe nausea was high 0–6 h and 6–24 h after surgery in high-risk patients (Apfel risk factors ≥ 3). Therefore, preventive strategies for PON and POV should aim to prevent symptoms in the late period (6–24 hours) as well as in the early period (0–6 hours). The difference in the effects of ramosetron and ondansetron in the early period was statistically significant, but we could not reach a firm conclusion because of the low quality of evidence (GRADE: low). Interpreting the results conservatively, we conclude that ramosetron had at least an equal effect to that of ondansetron in preventing early-period PON and POV. Thus, ramosetron could be recommended as a prophylactic drug for high-risk patients, since it was more effective in preventing PON and POV in the late period and was at least equally effective as ondansetron in the early period. However, cost-effectiveness studies comparing ramosetron and ondansetron should be conducted to support this strategy.

The GRADE score for the meta-analysis was “low” for early-period PON, early-period POV, and next-day POV. The main reason for the low GRADE score was imprecisions revealed by TSA. The TSA results for these outcomes showed that the cumulative Z-curve did not cross either the TSA monitoring boundary or the futility boundary, which means that the results could not lead to firm conclusions. The TSA also revealed that the acquired sample size reached only 30% in the next-day POV analysis, possibly due to a low incidence of next-day POV. A previous observational study [45] revealed that the incidence of next-day POV was low even in high-risk patients. Thus, next-day POV prevention may not be important when determining a prophylactic strategy for PON and POV.

The test for subgroup differences indicated that the dose of ondansetron influenced its efficacy on early-period PON. The effect of ramosetron in preventing early PON was greater than that of 4-mg ondansetron, but not of 8-mg ondansetron. When prescribing 8-mg ondansetron in routine clinical practice, some physicians may be concerned about the US Food and Drug Administration (FDA) alert regarding QT interval prolongation with high-dose ondansetron [46]. The FDA states that “the 32 mg, single IV dose should be avoided due to the risk of a specific type of irregular heart rhythm called QT interval prolongation, which can lead to Torsades de Pointes, an abnormal, potentially fatal heart rhythm” [46] and “no single intravenous dose should exceed 16 mg” [46]. Therefore, 8 mg of ondansetron could be considered for prophylaxis when the main target is to prevent PON in the early period (0–6 h after surgery).

We analyzed the side effects of 5-HT_3_ antagonists, including headache, dizziness, and drowsiness. The incidence of dizziness was significantly lower in patients receiving ramosetron than in patients receiving ondansetron (GRADE: low), but the incidences of headache and drowsiness did not differ for the two drugs (GRADE: low). We graded the quality of evidence of these outcomes as “low” because TSA revealed that the cumulative Z-curve did not cross the boundaries. More RCTs would be required to reach a firm conclusion regarding the comparative incidence of side effects of ramosetron and ondansetron.

There were limitations in our meta-analysis. First, the results of TSAs revealed that the acquired sample size did not reach the RIS (i.e., target sample size) in any primary outcome except for late PON. Although the results of late-period POV and next-day PON assessments showed that the cumulative Z-curve crossed the TSA monitoring boundary before reaching the RIS, the GRADE score was low for all other outcomes. To reach a firm conclusion, further RCTs are required to study the effects of the two drugs on early PON, early POV, and next-day POV. Second, we included only one RCT of pediatric patients in the meta-analysis. The overall risk of bias of the pediatric RCT was unclear, and thus, the RCT was excluded from our sensitivity analysis, restricting the analysis to high-quality RCTs. Therefore, our results cannot be extrapolated to pediatric patients.

In conclusion, the current meta-analysis shows that ramosetron is more effective at preventing late PON, late POV, and next-day PON than ondansetron, and may be associated with a lower incidence of dizziness.

## Supporting information

S1 Table The PRISMA checklist. (DOC)
